# Inhaled nitric oxide in patients admitted to intensive care unit with COVID-19 pneumonia

**DOI:** 10.1186/s13054-020-03222-9

**Published:** 2020-08-17

**Authors:** Guido Tavazzi, Pozzi Marco, Silvia Mongodi, Valentino Dammassa, Giovanni Romito, Francesco Mojoli

**Affiliations:** 1 Department of Clinical-Surgical, Diagnostic and Paediatric Sciences, Unit of Anaesthesia and Intensive Care, University of Pavia, Pavia, Italy; 2 Anesthesia and Intensive Care, Fondazione Policlinico San Matteo Hospital IRCCS, Anestesia e Rianimazione I, DEA Piano -1, Fondazione IRCCS Policlinico S. Matteo, Viale Golgi 19, 27100 Pavia, Italy

Dear Editor,

Patients with ARDS due to COVID-19 are characterised by poor oxygenation with a various extent of pulmonary alterations [1]. Ventilation strategies for COVID-19 patients have been suggested basing on the pathophysiological evidence to date [1]; however, there are no data regarding the use of inhaled nitric oxide (iNO). We report herein our experience of iNO administration in COVID-19 mechanically ventilated patients with refractory hypoxaemia and/or right ventricular (RV) dysfunction. Refractory hypoxaemia was defined as PaO_2_/FiO_2_ < 100 despite high PEEP (≥ 10 cmH_2_O) and prone position. RV dysfunction was defined as acute *cor pulmonale* at echocardiography with hemodynamic impairment requiring infusion of inotropic drugs [2].

The NO/nitrogen mixture was introduced into the inspiratory limb of the ventilator tubing. Respiratory and haemodynamic parameters were collected immediately before iNO administration (*t*_0_) and after 15–30 min (*t*_1_). Responders were defined by an increase of PaO_2_/FiO_2_ > 20% compared to *t*_0_ [3].

Results in the text are shown as median [IQR] or number (%). Wilcoxon test for paired samples and Mann-Whitney test, as appropriate (MedCalc version 19.2 MedCalc Software), were performed considering *p* < 0.05 as significant.

iNO was used in sixteen out of 72 (22.2%) consecutive mechanically ventilated patients (66.0 [59.6–69.7] years old; 93% male). All patients required iNO for refractory hypoxaemia of whom 4 (25%) had also superimposed RV dysfunction, in 1 case associated with pulmonary embolism. The iNO dosage was 25 [20–30] parts per million (ppm).

Respiratory parameters at *t*_0_ and *t*_1_ are shown in Table 1. Overall, iNO did not improve oxygenation in our population. Only 4 (25%) patients were responders, of whom 3 have superimposed RV dysfunction, showing a median increase of PaO_2_/FiO_2_ of 26.9% [24.1–45.5]. A trend towards a larger improvement of oxygenation was observed in patients with RV dysfunction as compared with those without (PaO_2_/FiO_2_ increase 24.1% [9.2–43.5] vs. 3.3% [− 10.8–11.5], *p* = 0.069). Additionally, in responders, PaO_2_/FiO_2_ was 125.9 [82.2–259.2] at *t*_1_ and did not change (*p* = 0.875) 24 h later (146.4 [102.2–225.1]).

**Table 1:** Patients respiratory and hemodynamic parameters at the two time points

Parameter	Pre iNO (***t***_**0**_)	Post iNO (***t***_**1**_)	***p*** value
SBP, mmHg	127.0 [114.0–137.5]	119.0 [110.0–138.0]	0.454
MAP, mmHg	83.5 [80.5–93.5]	78.0 [74.5–85.5]	0.144
HR, bpm	89.5 [80.5–99.7]	88.0 [75.0–100.0]	0.159
pH	7.27 [7.22–7.35]	7.31 [7.24–7.36]	0.049
PaCO_2_, mmHg	59.8 [52.5–76.5]	60.9 [50.8–65.7]	0.002
PaO_2_, mmHg	79.7 [58.9–87.2]	77.1 [63.5–88.6]	0.252
PaO_2_/FiO_2_	91.7 [62.1–109.2]	91.5 [67.1–106.7]	0.274
MetHb, %	1.18 [1–1.3]	1.3 [1.1–1.4]	0.16
FiO_2_	87.5 [80–95]	87.5 [80–95]	1
PEEP, cmH_2_O	13.0 [10.0–15.0]	13.0 [10.0–15.0]	1
MV, L/min	9.7 [8.1–11.3]	10.3 [8.7–11.4]	0.204
Peak pressure, cmH_2_O	30.5 [27.5–33.5]	30.5 [26.0–33.0]	0.641

Results in the table are shown as mean [CI 95%]

*SBP* systolic blood pressure, *MAP* mean arterial pressure, *HR* heart rate, *bpm* beats per minutes, *MetHb* methemoglobin, *PEEP* positive end-expiatory pressure, *MV* minute volume

iNO is a free radical gas that diffuses across the alveolar-capillary membrane into the subjacent smooth muscle of pulmonary vessels enhancing endothelium-dependent vasorelaxation and improving oxygenation by increasing blood flow to ventilated lung units [3]. In previous studies, iNO was effective in improving PaO_2_/FiO_2_ and oxygenation index, although it failed in reversing acute lung injury, reducing mechanical ventilation days and mortality [4].

In our population, the improvement of oxygenation in responders was probably magnified by an iNO-induced decrease of RV afterload, enhancing cardiac output and finally leading to an increase of mixed venous oxygen saturation.

Although the reason why patients with refractory hypoxaemia without RV dysfunction were not responder is yet to be determined, some speculation can be done. Severe endothelial injury with cytoplasmic vacuolization and cell detachment in pulmonary middle-small arteries can make the pulmonary vessels less reactive to iNO stimulation [1, 5, 6]. This could also explain the loss of hypoxic vasoconstriction and lung perfusion regulation. However, whether vascular derangements in COVID-19 are due to endothelial cell involvement by the virus, part of the ARDS pathophysiology or the intertwine of both is still undetermined. Moreover, prone position and iNO were used in refractory hypoxaemia as an escalating treatment strategy. Therefore, a positive response to the prone position may have precluded the enrolment in our study of patients that could positively respond to iNO.

## Conclusion

Overall, iNO did not improve oxygenation in COVID-19 patients with refractory hypoxaemia, when administered as a rescue treatment after prone position. A subgroup of patients with RV dysfunction was better iNO responders probably due to the haemodynamic improvement associated with RV unloading.

*The word count of our manuscript is just beyond the limit suggested by the editorial rules as we felt that the fluency and completeness would be sacrificed in further shorten the text. However, we are willing to cut some part if strongly advised by the editorial office.*

## Data Availability

The datasets used and/or analysed during the current study are available from the corresponding author on reasonable request.
