# Correction to: Inhaled nitric oxide in patients admitted to intensive care unit with COVID-19 pneumonia

**DOI:** 10.1186/s13054-020-03390-8

**Published:** 2020-11-26

**Authors:** Guido Tavazzi, Marco Pozzi, Silvia Mongodi, Valentino Dammassa, Giovanni Romito, Francesco Mojoli

**Affiliations:** 1 Department of Clinical-Surgical, Diagnostic and Paediatric Sciences, Unit of Anaesthesia and Intensive Care, University of Pavia, Pavia, Italy; 2 Anesthesia and Intensive Care, Fondazione Policlinico San Matteo Hospital, IRCCS, Anestesia e Rianimazione I, DEA Piano -1, Fondazione IRCCS, Policlinico S. Matteo, Viale Golgi 19, 27100 Pavia, Italy

## Correction to: Critical Care (2020) 24:508 https://doi.org/10.1186/s13054-020-03222-9

Following publication of the original article [1], the authors identified an error in an author name. The given name and family name were erroneously transposed for Marco Pozzi.

The incorrect author name is:

Pozzi Marco

The correct author name is:

Marco Pozzi

The author name has been updated above and the original article [1] has been corrected.
